# Detection of *Leishmania* and *Trypanosoma* DNA in Field-Caught Sand Flies from Endemic and Non-Endemic Areas of Leishmaniasis in Southern Thailand

**DOI:** 10.3390/insects10080238

**Published:** 2019-08-02

**Authors:** Pimpilad Srisuton, Atchara Phumee, Sakone Sunantaraporn, Rungfar Boonserm, Sriwatapron Sor-suwan, Narisa Brownell, Theerakamol Pengsakul, Padet Siriyasatien

**Affiliations:** 1 Medical Parasitology Program, Department of Parasitology, Faculty of Medicine, Chulalongkorn University, Bangkok 10330, Thailand; 2 Vector Biology and Vector Borne Disease Research Unit, Department of Parasitology, Faculty of Medicine, Chulalongkorn University, Bangkok 10330, Thailand; 3 Thai Red Cross Emerging Infectious Diseases-Health Science Centre, World Health Organization Collaborating Centre for Research and Training on Viral Zoonoses, Chulalongkorn Hospital, Bangkok 10330, Thailand; 4 Medical Science Program, Faculty of Medicine, Chulalongkorn University, Bangkok 10330, Thailand; 5 Faculty of Medical Technology, Prince of Songkla University, Songkhla 90110, Thailand

**Keywords:** *Leishmania* spp., *Trypanosoma* sp., sand flies, vector, Thailand

## Abstract

Phlebotomine sand flies are tiny, hairy, blood-sucking nematoceran insects that feed on a wide range of hosts. They are known as a principal vector of parasites, responsible for human and animal leishmaniasis worldwide. In Thailand, human autochthonous leishmaniasis and trypanosomiasis have been reported. However, information on the vectors for *Leishmania* and *Trypanosoma* in the country is still limited. Therefore, this study aims to detect *Leishmania* and *Trypanosoma* DNA in field-caught sand flies from endemic areas (Songkhla and Phatthalung Provinces) and non-endemic area (Chumphon Province) of leishmaniasis. A total of 439 sand flies (220 females and 219 males) were collected. Head and genitalia dissection of female sandflies were done for morphology identification, and the remaining parts of those sand flies were then used for the detection of *Leishmania* and *Trypanosoma* parasites. The DNA was extracted from individual female sand flies. Polymerase chain reaction (PCR) anneal, specific to the *ITS1* and *SSU rRNA* gene regions, was used to detect *Leishmania* and *Trypanosoma* DNA, respectively. The positive PCR products were cloned and sequenced. The results showed that the female sand fly species in this study consisted of *Sergentomyia khawi* (35.9%); *Se. anodontis* (23.6%); *Phlebotomus betisi* (18.6%); *Ph. kiangsuensis* (9.5%); *Ph. asperulus* (6.4%); *Se. barraudi* (2.3%); 0.9% of each *Se. indica*, *Ph. stantoni*, and *Ph. major major*; and 0.5% of each *Se. sylvatica* and *Ph. mascomai*. The PCR and sequence analysis were able to detect *Leishmania* and *Trypanosoma* DNA in sand fly samples, which were identified as *L. martiniquensis*, 1/220 (0.45%) in *Se. khawi*, 3/220 (1.36%) of *T. noyesi* in *Se. anodontis*, and *Ph. asperulus.* Fourteen (6.36%) of the unidentified trypanosome species in *Se. khawi*, *Se. indica*, *Se. anodontis*, *Ph. asperulus*, and *Ph. betisi* were found in all of the areas of this study. Interestingly, we found a 1/220 (0.45%) co-infection sample of *L. martiniquensis* and *Trypanosoma* in *Se. khawi* from Songkhla Province. These data indicate that several species of sand flies might be potential vectors of *Leishmania* and *Trypanosoma* parasites in southern Thailand. However, more extensive study for potential vectors using a larger number of sand flies should be conducted to prove whether these sand flies can be natural vectors of leishmaniasis and trypanosomiasis in both humans and animals. In addition, our study could be useful for the future study of infection prevention, including effective vector control for leishmaniasis and trypanosomiasis in Thailand.

## 1. Introduction

Phlebotomine sand flies are small dipteran, hairy, and blood-sucking insects belonging to the order Diptera and family Psychodidae [1], which are known as important medical vectors of leishmaniasis worldwide. Leishmaniasis is a neglected tropical disease caused by the flagellate protozoa of the genus *Leishmania* [2]. Moreover, Phlebotomine sand flies have been reported to be the potential vector of the *Trypanosoma* species, which can cause trypanosomiasis in mammals, birds, fish, rats, sheep, cows, rabbits, lizards, frogs, and snakes [3,4,5,6,7]. Both parasite genera have been reported in many areas of the world. In Thailand, *Leishmania orientalis* [8], *L. martiniquensis* [9], and *L. donovani* complex [10,11] have been identified as the causative agents of indigenous visceral and cutaneous leishmaniasis. There have been approximately 20 autochthonous leishmaniasis reported in the country from 1996 to the present [12]. The disease is endemic mostly in southern Thailand. Trypanosomiasis is commonly found in animals, for example, *T. lewisi* and *T. evansi*. These animals are the major pathogenic trypanosome of domestic animals, such as rodents, cattle, wild animals, elephants, and tigers [13]. Furthermore, *T. lewisi* [14] and *T. lewisi*-like infection [15] have been reported in an infant in Thailand. It has been almost 20 years since the first case of autochthonous leishmaniasis was reported in Thailand [10], but data of the sand fly vector for the infectious diseases are still limited. In Thailand, several reports of sand flies in the western, central, northern, southern, and northeastern regions have identified four genera of sand flies, as follows: *Sergentomyia*, *Phlebotomus*, *Idiophlebotomus*, and *Chinius* [16,17,18,19,20]. The genus *Sergentomyia* was mostly demonstrated in the Phangnga, Suratthani, and Nakonsitammarat Provinces of southern Thailand, which are considered to be the affected areas of leishmaniasis. Sand fly species, such as *Sergentomyia gemmea* (81.4%), *Se. iyengari*, *Se. barraudi*, *Se. indica*, *Se. sylvatica*, and *Se. perturbans*, as well as another genus, *Phlebotomus* (*Ph. argentipes*), are found in the aforementioned affected areas in the country [21]. Moreover, *Se. (Neophlebotomus) gemmea* collected from an endemic area of leishmaniasis in Thailand were detected for *L. martiniquensis* DNA; therefore, *Se. gemmea* was claimed to be a potential vector of *L. martiniquensis* [22]. Recently, Phumee et al. (2016) reported an unknown *Trypanosoma* sp. DNA detected in a *Ph. stantoni* from Thailand [23]. However, the information on other sand fly species, which may transmit leishmaniasis and trypanosomiasis, is also limited. In this study, we focused on the detection of *Leishmania* and *Trypanosoma* parasites in sand flies collected from endemic and non-endemic areas of leishmaniasis in southern Thailand. The information from this study might help us to gain a better understanding about the prevalence of parasites in the sand fly population, and about the association among the sand fly species as a potential vector in endemic areas of leishmaniasis in Thailand.

## 2. Materials and Methods 

### 2.1. Ethics Statement

The study was approved by the animal research ethics committee of Chulalongkorn University Animal Care and Use Protocol (CU-ACUP), Faculty of Medicine, Chulalongkorn University, Bangkok, Thailand (COA No. 019/2561).

### 2.2. Sand Fly Collection, Sample Preparation, and Identification

**Figure 1:**
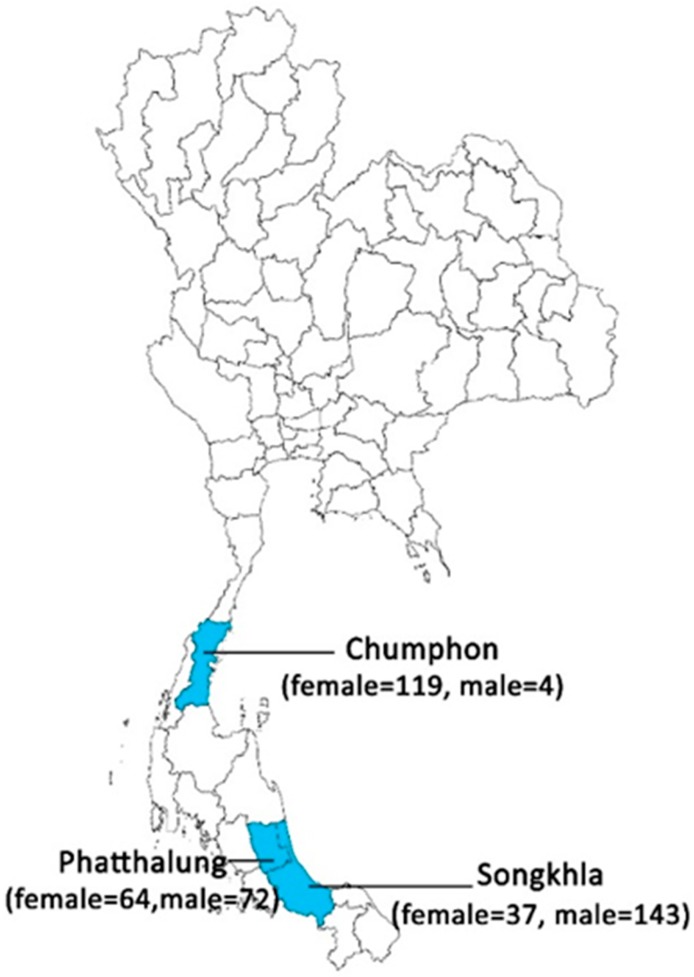
Map of Thailand showing locations of the sand fly sample-collection sites in the three provinces. Blue denotes the collection locations of sand flies.

Sand flies were collected from endemic areas, around human settlements (Songkhla and Phatthalung Provinces), and non-endemic areas, in caves (Chumphon Provinces), of leishmaniasis in southern Thailand (Figure 1). The sand fly specimens were collected by Centers for Disease Control and Prevention (CDC) miniature light traps (25 W bulb) without CO_2_, during September–October 2017. The 10 traps operated for over 12 h, from 18:00 to 06:00, for one night. The collecting bags were kept at −20 °C for 30 min in order to anesthetize the arthropods and separate the sand flies from the other arthropods by morphological features using a stereomicroscope (Olympus, Tokyo, Japan). Each female sand fly was dissected under a stereomicroscope on a sterilized slide using sterile needles. The head and genitalia were mounted on slides with Hoyer’s medium for morphological character identification in all female sand flies, under a light microscope (Olympus, Tokyo, Japan), following the taxonomic keys described by Lewis (1978) [24], Phumee et al. (2016) [23], and Depaquit et al. (2019) [25]. The abdomen and the thorax of each female specimen were transferred to sterile 1.5 mL Eppendorf tubes with a lysis buffer for the detection of *Leishmania* and *Trypanosoma* species by the polymerase chain reaction (PCR).

### 2.3. DNA Extraction 

DNA was extracted from individual sand fly samples using an Invisorb Spin Tissue Mini Kit (STRATEC Molecular, Berlin, Germany), following the manufacturer’s instructions. The sand flies were lysed in a 200 µL lysis buffer containing 20 µL of proteinase K. At the final step, DNA was eluted in a 40 µL of elution buffer. The extracted DNA samples were kept at −80 °C for long-term storage.

### 2.4. Detection of Leishmania and Trypanosoma DNA in the Sand Fly Samples

The extracted DNA samples were used to detect the *Leishmania* DNA using polymerase chain reaction (PCR) amplification annealed specifically to the *ITS1* region. The reactions were performed using primers LeR: 5′-CCA-AGT-CAT-CCA-TCG-CGA-CAC-G-3′ and LeF: 5′-TCC-GCC-CGA-AAG-TTC-ACC-GAT-A-3′, under the conditions previously described by Spanakos et al. (2008) [26]. The reaction was carried out in a total volume of 25 μL, containing 2.5 μL of 10× PCR Buffer (NH_4_SO_4_), 1.25 μL of 25 mM MgCl_2_, 2.5 μL of 2.5 mM of each dNTP, 10 mM of each primer, 1 unit of *Taq* DNA polymerase, and 50 ng of DNA template. After initial denaturation at 95 °C for 5 min, PCR was performed, with 40 cycles consisting of denaturation (95 °C for 1 min), annealing (50 °C for 1 min), extension (72 °C for 1 min), and a final extension at 72 °C for 7 min. The PCR products were analyzed by electrophoresis on a 1.5% agarose gel for 40 min at 100 volts, stained with ethidium bromide, and visualized with Quantity One Quantification Analysis Software Version 4.5.2 (Gel DocEQ System; Bio-Rad, Hercules, CA, USA). For the identification of the *Trypanosoma* species, the small subunit ribosomal RNA (*SSU rRNA*) region was amplified from the sand fly using primers designed specifically for trypanosomatids. The sequences primers were TRY927F: 5′-GAA-ACA-AGA-AAC-ACG-GGA-G-3′ and TRY927R: 5′-CTA-CTG-GGC-AGC-TTG-GA-3′ [27]. The reaction and conditions followed the amplification of the *ITS1* region described previously.

### 2.5. Molecular Cloning and Nucleotide Sequencing

The amplified PCR products were cloned into the pGEM-T Easy Vector (Promega, Madison, WI, USA). The ligated vectors were transformed into *E. coli* DH5∝ competent cells. The recombinant plasmids were screened using the blue-white screening systems. Suspected chimeric colonies were cultured and subjected to plasmid DNA extraction by the Invisorb Spin Plasmid Mini Kit (STRATEC Molecular, Berlin, Germany), following the manufacturer’s instructions. The purified plasmids were sequenced by the sequencing service of Macrogen Inc., Seoul, Korea, using a universal forward T7 primer. The nucleotide sequences were analyzed by comparison with the GenBank database using a BLAST search (https://blast.ncbi.nlm.nih.gov/Blast.cgi). The sequences were aligned using BioEdit Sequence Alignment Editor Version 7.0.5.3 [28]. The phylogenetic tree was constructed using the maximum likelihood method with Kimura’s two-parameter and bootstrap analysis with 1000 replications in MEGAX version 10.0.1 [29]. 

## 3. Results

The number of sand flies collected from all of the sites (Songkhla, Phatthalung, and Chumphon Provinces) using a CDC light trap was 439 (220 females and 219 males). For the morphological characteristics, the female sand flies (n = 220) were classified into two genera and 11 species, which were from the Songkhla (*Se. anodontis*, *Se. khawi*, *Se. barraudi*, and *Ph. stantoni*), Phatthalung (*Se. khawi*, *Se. barraudi*, *Se. indica*, *Ph. betisi*, and *Ph. kiangsuensis*), and Chumphon (*Se. anodontis*, *Se. sylvatica*, *Ph. asperulus*, *Ph. betisi*, *Ph. kiangsuensis*, *Ph. major major*, and *Ph. mascomai*) provinces. The most abundant species in our samples was *Se. khawi* (35.9%; Table 1). All of the samples of female sand flies were screened for *Leishmania* and *Trypanosoma* infection using *ITS1*-PCR and *SSU rRNA*-PCR, respectively.

In the endemic area of leishmaniasis, Songkhla province, one out of thirty-seven female *Se. khawi* DNA extracts was positive, by PCR, for *L. martiniquensis* DNA, using *ITS1*-PCR (Figure S1), and one sample showed co-infection between *L. martiniquensis* and *Trypanosoma* parasites (Table 2). An analysis of the *L. martiniquensis* amplified sequences showed 99% compatibility with *L. martiniquensis* (accession no. JQ001751; Figure S2). Moreover, PCR targeting the *SSU rRNA* region of the four positive samples showed 99% identity with *Trypanosoma* sp. (accession no. AB520638; Figure S3). The female sand flies collected from Phatthalung were 50 sand flies of *Se. khawi* and two sand flies of *Se. indica*. One positive sample showed a 99% similarity with *Trypanosoma* sp. (accession no. AB520638) in each species, whereas *Leishmania* DNA was not detected in this area (Figure S4).

The female sand flies were collected from the non-endemic area of leishmaniasis, Chumphon Province. The results showed that 2/119 of *Se. anodontis* and 1/119 of *Ph. asperulus* were detected for *T. noyesi* DNA, with 98% identity (accession no. KX008320); furthermore, we found positive *Trypanosoma* sp. in four samples of *Se. anodontis* and two samples of each *Ph. asperulus* and *Ph. betisi*, which showed 96%–99% similarity with *Trypanosoma* sp. (accession no. AB520638). However, *Leishmania* DNA was not detected in the non-endemic area of leishmaniasis.

**Figure 2:**
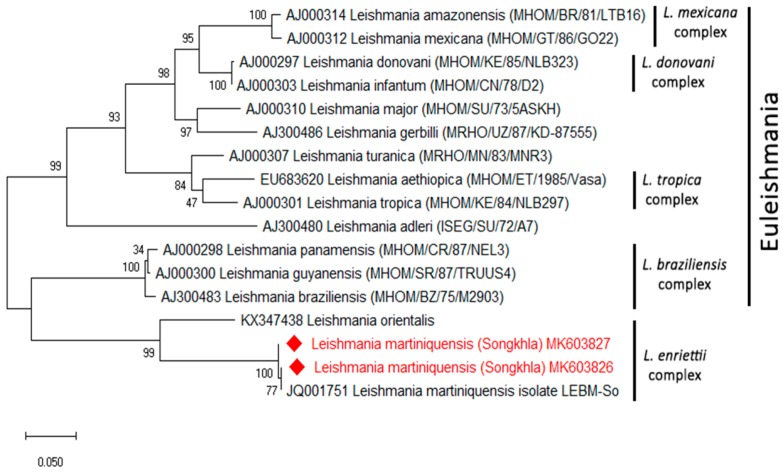
A maximum likelihood tree was constructed from the partial *ITS1* sequences of *Leishmania* spp. and was compared with the reference sequences using the Kimura two-parameter model with the maximum likelihood method, by testing with 1000 bootstrap values. The sequences from this study are indicated with a red color.

**Figure 3:**
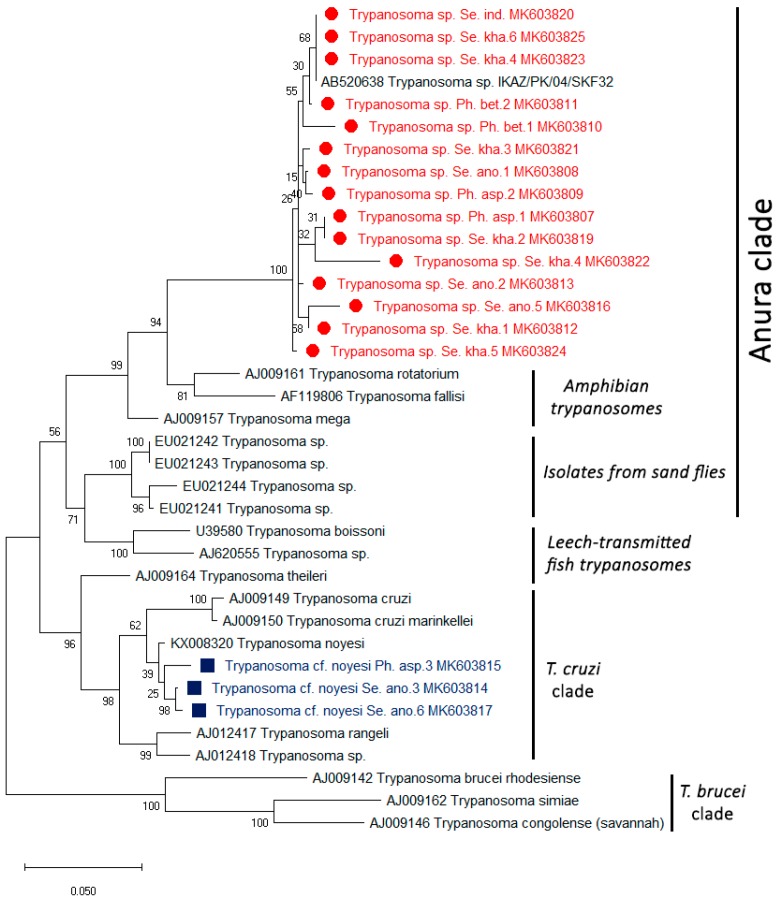
Phylogenetic tree of the *Trypanosoma* species in the sand flies constructed from the partial *SSU rRNA* sequences from all of the regions. The sequences from this study, indicated in red and blue, were compared with the reference sequences obtained from GenBank. The tree was derived using the maximum likelihood method based on the Kimura two-parameter model (bootstrapped 1000 times).

The phylogenetic tree of *L. martiniquensis* of the *ITS1* region was clearly shown in a clade of the *L. enriettii* complex (Figure 2). The phylogenetic tree of all of the positive *Trypanosoma* parasites with the *SSU rRNA* regions was divided into two groups. The first group of 15 samples contained *Trypanosoma* sp. in the Anura clade described in Pakistan. The second group of three samples was grouped together with *T. noyesi* with small branch lengths in the *T. cruzi* clade (Figure 3). The partial nucleotide sequence of the *ITS1* and *SSU rRNA* obtained in this study was deposited in GenBank under the following accession numbers: MK603807–MK603827.

## 4. Discussion

Autochthonous leishmaniasis in Thailand is caused by two major *Leishmania* species, namely, *L. orientalis* and *L. martiniquensis* (Kinetoplastida: Trypanosomatidae) [8,9,12,30,31,32,33,34,35,36,37,38,39,40]. Studies on the vector of the disease are essential for controlling the disease and for gaining a better understanding of the disease transmission dynamics; however, there are limited data on this disease in Thailand. Ready (2013) has demonstrated that female sand flies are able to transmit several parasites, including *Leishmania* spp., *Trypanosoma* spp., and other viruses [41]. In the current study, we described a method for the detection of *Leishmania* and *Trypanosoma* protozoans by PCR using primers specific for *Leishmania* and *Trypanosoma* DNA in a natural population of sand flies collected from endemic and non-endemic areas of leishmaniasis in southern Thailand. We showed that the only *L. martiniquensis*-positive sand flies were *Se. khawi*, by analysing the *ITS1* regions. Moreover, one sample showed co-infection between *L. martiniquensis* and *Trypanosoma* sp. DNA in *Se. khawi* (*Trypanosoma* sp. *Se. kha*) from the Songkhla Province, an endemic area of leishmaniasis. However, we did not conduct a dissection to demonstrate live parasites in the midgut and did not observe any blood meal in the female sand fly samples; however, we also detected parasite DNA in the samples. These results could infer that there may be some live parasites in the midgut, even after the female sand flies have been defecated. In the next survey study, we will dissect the sand fly midguts for the live parasites. The have been reports of the potential role of *Sergentomyia* species as a vector of leishmaniasis [42]. *L. donovani* DNA has been detected in *Se. babu* from India [43]. *L. major* DNA has been detected in *Se. sintoni* from Iran [44], *Se. minuta* from Portugal [45], and *Se. clydei* [46] and *Se. minuta* [47] from Tunisia. In addition, *L. infantum* DNA has been detected in *Se. dubia*, *Se. magna*, and *Se. schwetzi* from Senegal [48]. In Thailand, *L. martiniquensis* DNA was detected in both *Se. gemmea* [12,22] and *Se. barraudi* [49]. Recently, Siripattanapipong et al. (2018) suggested that *L. orientalis* was found in *Se. iyengari* (Diptera: Psychodidae) from southern Thailand [50]. However, some reports have described that the species of *Se. gemmea* and *Se. iyengari* sand flies in Thailand may be misidentified based on morphology and molecular techniques [23,25]; moreover, all of the sequences of *Se. iyengari* deposited in GenBank (accession numbers MG770900, MG770901, MG770913, MG770914, MG770916–MG770918, MG770924, MG770926, and MG770927) and *Se. gemmea* deposited in GenBank (accession numbers MG770900, MG770901, MG770913, MG770914, MG770916–MG770918, MG770924, MG770926, and MG770927) are grouped as *Se. hivernus* (Raynal and Gaschen) and *Se. khawi* (Raynal) by using *CytB*-PCR [23,25]. In this study, the most abundant species in our samples was *Se. khawi*, which could be a potential vector of *L. martiniquensis* infection in both humans and animals in Thailand. However, in order to prove that *Se. khawi* is a competent vector of leishmaniasis, future studies are needed in order to reveal exact sand fly species in both the morphological and molecular characterization. Additionally, the isolation and cultivation of *Leishmania* parasites are needed to support the complete life cycle of leishmaniasis in Thailand. 

For *Trypanosoma* DNA detection, we found 1.81% of *T. noyesi* in *Se. anodontis* (*Trypanosoma* cf. *noyesi Se. ano*) and *Ph. asperulus* (*Trypanosoma* cf. *noyesi Se. asp*) and 6.36% of *Trypanosoma* sp. *Se. kha*, *Se. indica* (*Trypanosoma* sp. *Se. ind*), *Se. anodontis* (*Trypanosoma* sp. *Se. ano*), *Ph. asperulus* (*Trypanosoma* sp. *Ph. asp*), and *Ph. betisi* (*Trypanosoma* sp. *Ph. bet*) from both endemic and non-endemic regions of leishmaniasis. Three positive samples were clustered together with *T. noyesi* (accession no. KX008320) with small branch lengths and in the *T. cruzi* clade. Botero et al. (2016) determined that their study provided novel information on the morphological, behavioral observations in vitro, and genetic variability of an Australian trypanosome of *T. noyesi* within the *T. cruzi* clade isolated from the critically endangered woylie (*Bettongia pencillata*) [51]. A previous report in Thailand was the first study on *Trypanosoma* sp. DNA detection in a *Ph. stantoni* collected from southern Thailand. It was suspected that the detected sequences belonged to a novel species of the genus *Trypanosoma* [23]. In this study, we found that 15 samples were grouped to *Trypanosoma* sp. from *Ph. Kazeruni*, and Theodor and Mesghali described this in Pakistan (accession no. AB520638), in an Anura clade [52]. In contrast, we have not found unknown *Trypanosoma* sp. in sand flies, as described by Phumee et al., (2016) in Thailand. This might be because of the low number of samples, and the vector for unknown *Trypanosoma* sp. may be other sand fly species. Many reports also described the detection of *Trypanosome* DNA in sand flies. For example, Nzelu et al. (2014) revealed that *L. tropica* and *L. major* DNA, and *Trypanosoma* DNA can be detected in *Sergentomyia* sand flies in Ghana [53]. However, the information of *Leishmania* and *Trypanosoma* parasites in sand flies associated with their hosts, geographic distribution, and human or animal diseases are quite limited. Therefore, more extensive and systematic epidemiological surveys on potential vectors by using a larger number of sand flies should be conducted for proving whether these sand flies can be natural vectors of leishmaniasis and trypanosomiasis in both humans and animals. In addition, a better understanding of the epidemiological and ecological relationships of the disease could be used for studying the prevention, as well as effective control for both *Leishmania* and *Trypanosoma* vectors in Thailand.

## 5. Conclusions

The natural infection of sand flies by *Leishmania* or *Trypanosoma* alone and the co-infection of both parasites were detected in both the *Sergentomyia* and *Phlebotomus* genera of sand flies. The detection and identification of natural infections in sand flies by these parasites in endemic and non-endemic areas of Thailand are key factors in assessing the disease transmission risk and potential vectors, designing prevention and control measures, and predicting disease epidemics in leishmaniasis and trypanosomiasis in Thailand. 

## Figures and Tables

**Table 1:** Morphological identification of female sand flies from southern Thailand.

Species	Provinces			Total
	Songkhla	Phatthalung	Chumphon	
*Se. anodontis*	4	0	48	52
*Se. khawi*	29	50	0	79
*Se. sylvatica*	0	0	1	1
*Se. barraudi*	2	3	0	5
*Se. indica*	0	2	0	2
*Ph. stantoni*	2	0	0	2
*Ph. asperulus*	0	0	14	14
*Ph. betisi*	0	3	38	41
*Ph. kiangsuensis*	0	6	15	21
*Ph. major major*	0	0	2	2
*Ph. mascomai*	0	0	1	1
**Total**	**37**	**64**	**119**	**220**

**Table 2:** Molecular detection of *Leishmania* DNA of *ITS1* and *Trypanosoma* DNA of *SSU rRNA* from sand fly samples collected in southern Thailand.

Province	Positive Sand Fly Species	Detection of *Leishmania* spp. and *Trypanosoma* sp.		
		*L. martiniquensis*	*T. noyesi*	*Trypanosoma* sp.
**Songkhla** **(n = 37)**	*Se. khawi*	2 *	ND	4
**Phatthalung** **(n = 64)**	*Se. khawi*	ND	ND	1
	*Se. indica*	ND	ND	1
**Chumphon** **(n = 119)**	*Ph. asperulus*	ND	1	2
	*Se. anodontis*	ND	2	4
	*Ph. betisi*	ND	ND	2
**Total (n = 220)**		1	3	14

* One specimen was co-infected by *L. martiniquensis* and *Trypanosoma* sp.; ND—not detected.

## Supplementary Materials

The following are available online at https://www.mdpi.com/2075-4450/10/8/238/s1. Figure S1: *ITS1*-PCR amplification for *Leishmania* parasites. Lane M: molecular mass marker (100 base pairs [bp]); lane P: *L. martiniquensis* culture, lane N: negative control, lanes 1–10 female sand fly samples. Figure S2: Sequence alignment of *Leishmania* parasites based on the partial *ITS1* gene. Figure S3: *SSU rRNA*-PCRs for *Trypanosoma* parasites. Lane M: molecular mass marker (100 base pairs [bp]); lane P: *Trypanosoma* sp. culture, lane N: negative control, lanes 1–10 female sand fly samples. Figure S4: Sequence alignment of *Trypanosoma* parasites base on the partial *SSU rRNA* gene.
