# Supplementary figures and images for: Detection of Leishmania and Trypanosoma DNA in Field-Caught Sand Flies from Endemic and Non-Endemic Areas of Leishmaniasis in Southern Thailand
Source: Insects. 2019 Aug 2;10(8):238. doi: 10.3390/insects10080238 (PMC6722825; doi:10.3390/insects10080238)

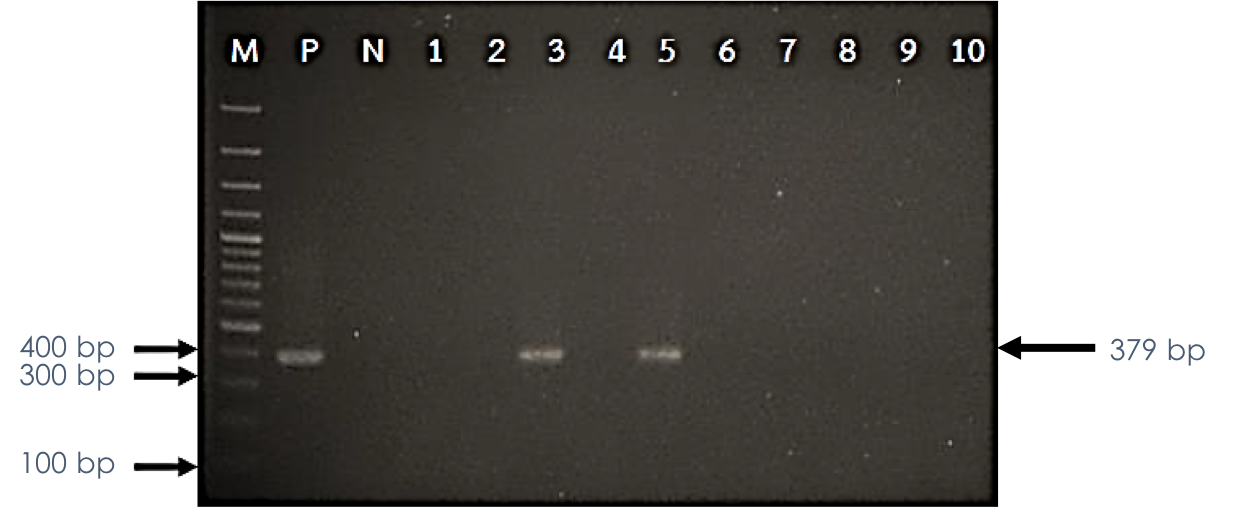

Supplement: Supplementary file 1 [file insects-10-00238-s001.zip › supplements/Figure S1.png]

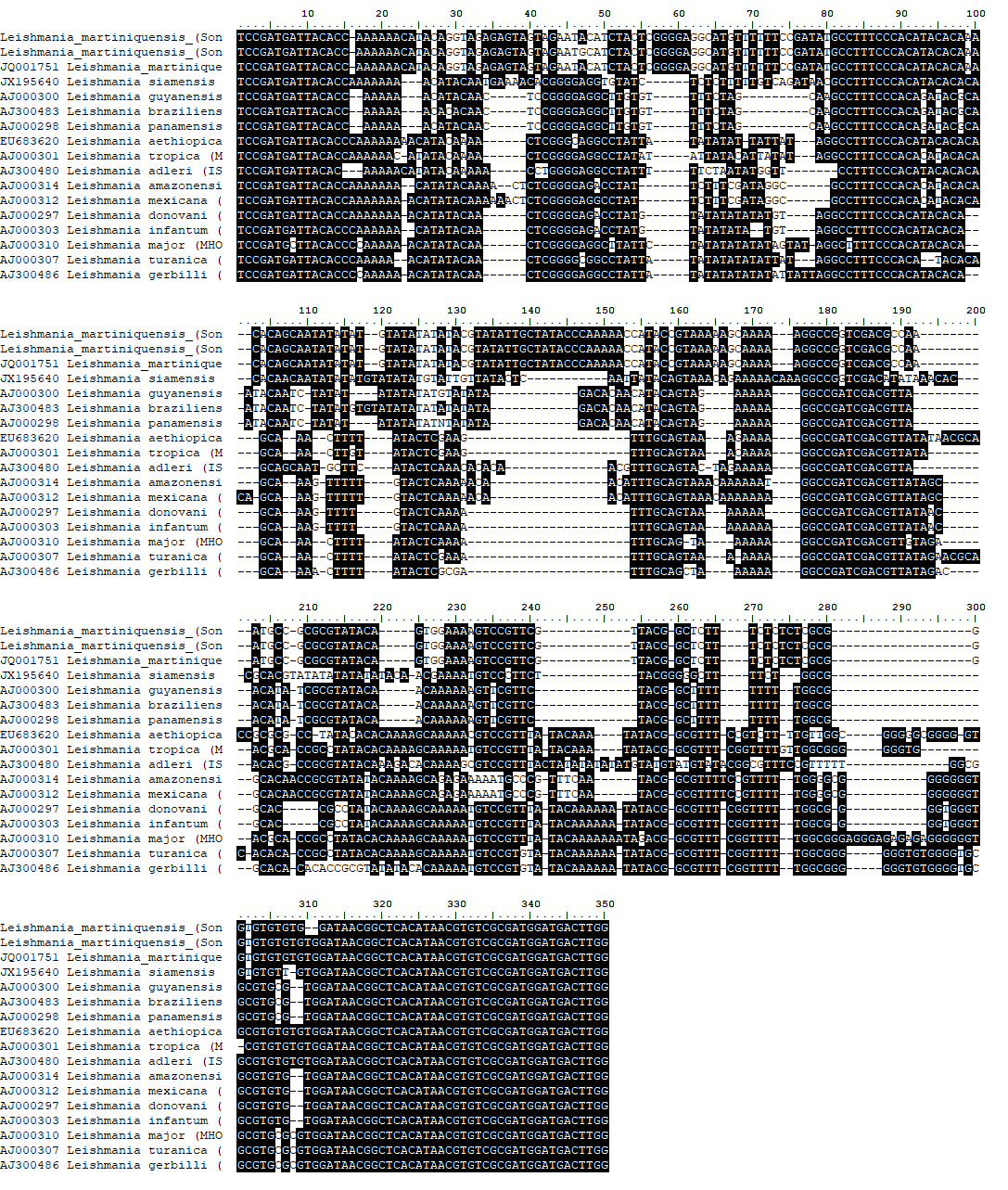

Supplement: Supplementary file 1 [file insects-10-00238-s001.zip › supplements/Figure S2.png]

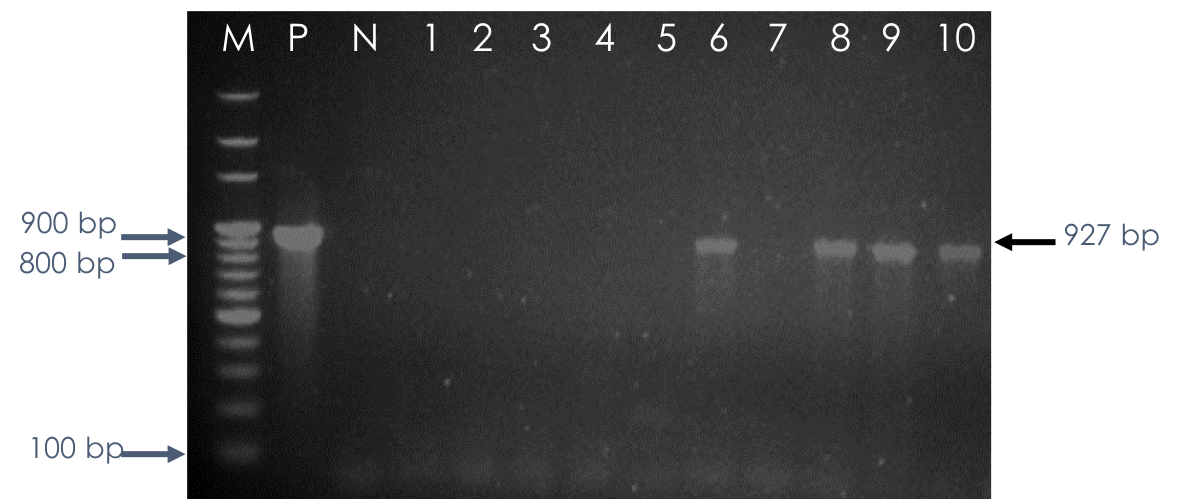

Supplement: Supplementary file 1 [file insects-10-00238-s001.zip › supplements/Figure S3.png]

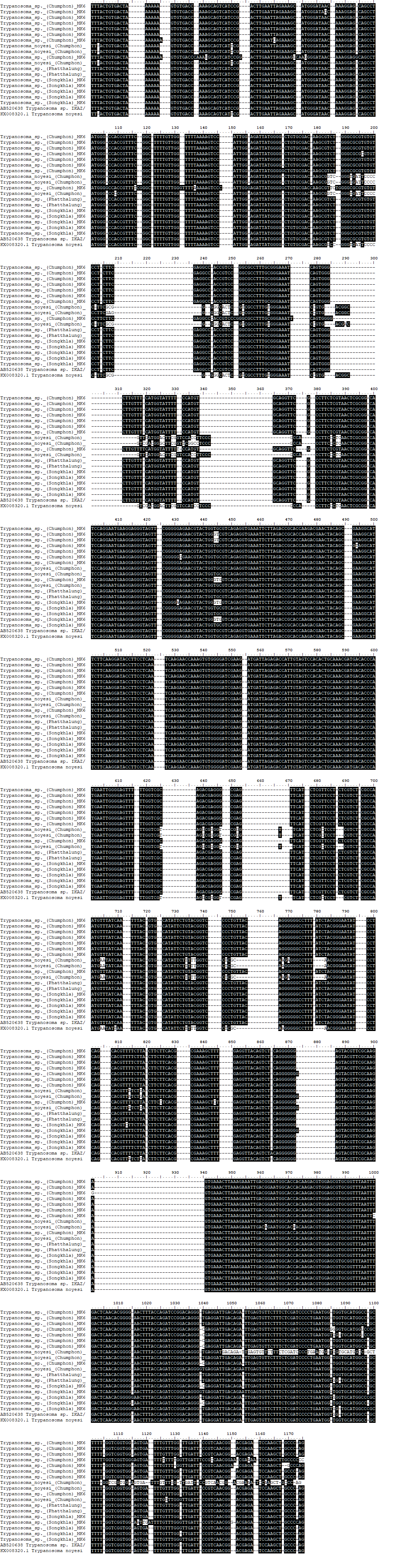

Supplement: Supplementary file 1 [file insects-10-00238-s001.zip › supplements/Figure S4.png]
